# Electrochemically Synthesis of Nickel Cobalt Sulfide for High‐Performance Flexible Asymmetric Supercapacitors

**DOI:** 10.1002/advs.201700375

**Published:** 2017-12-02

**Authors:** Chunyan Zhang, Xiaoyi Cai, Yao Qian, Haifeng Jiang, Lijun Zhou, Baosheng Li, Linfei Lai, Zexiang Shen, Wei Huang

**Affiliations:** ^1^ Key Laboratory of Flexible Electronics & Institute of Advanced Materials (IAM) Jiangsu National Synergistic Innovation Center for Advanced Materials (SICAM) Nanjing Tech University 5 XinMofan Road Nanjing 210009 China; ^2^ School of Physical and Mathematical Sciences Nanyang Technological University 21 Nan‐yang Link Singapore 637371 Singapore; ^3^ Key Laboratory of Organic Electronics & Information Displays (KLOEID) & Institute of Advanced Materials (IAM) Jiangsu National Synergetic Innovation Center for Advanced Materials (SICAM) Nanjing University of Posts & Telecommunications 9 Wenyuan Road Nanjing 210023 China

**Keywords:** asymmetric supercapacitors, electrochemical deposition, flexible electrodes

## Abstract

A lightweight, flexible, and highly efficient energy management strategy is highly desirable for flexible electronic devices to meet a rapidly growing demand. Herein, Ni–Co–S nanosheet array is successfully deposited on graphene foam (Ni–Co–S/GF) by a one‐step electrochemical method. The Ni–Co–S/GF composed of Ni–Co–S nanosheet array which is vertically aligned to GF and provides a large interfacial area for redox reactions with optimum interstitials facilitates the ions diffusion. The Ni–Co–S/GF electrodes have high specific capacitance values of 2918 and 2364 F g^−1^ at current densities of 1 and 20 A g^−1^, respectively. Using such hierarchical Ni–Co–S/GF as the cathode, a flexible asymmetric supercapacitor (ASC) is further fabricated with polypyrrple(PPy)/GF as the anode. The flexible asymmetric supercapacitors have maximum operation potential window of 1.65 V, and energy densities of 79.3 and 37.7 Wh kg^−1^ when the power densities are 825.0 and 16100 W kg^−1^, respectively. It's worth nothing that the ASC cells have robust flexibility with performance well maintained when the devices were bent to different angles from 180° to 15° at a duration of 5 min. The efficient electrochemical deposition method of Ni–Co–S with a preferred orientation of nanosheet arrays is applicable for the flexible energy storage devices.

## 1 Introduction

Flexible electronics are highly desirable as they offer the promise of readily customizable, and wearable electronics along with weight and volume savings.^1^ The flexible energy storage unit with high energy and power density represents an indispensable component for flexible electronics and has attracted significant interests. Supercapacitors are promising high power energy storage devices due to their long cycle life, low maintenance, high rates, and short response time.^2^ However, the commercially available supercapacitor electrodes are mainly based on activated carbon, which needs the addition of binders and conductivity enhancers and is challenging to achieve high flexibility and light weight. The ideal flexible electrodes should have energy density values close to those conventional supercapacitors, and its robust flexibility does not undermine other electrochemical characteristics, such as cycle life and rate performance.^3^ Although there are vast reports of carbon fiber paper,^4^ carbon cloth,^5^ metal mesh,^6^ CNT paper^7^ for flexible supercapacitors, these current collectors have high areal density range from ≈11.9 mg cm^−2^ (0.15 mm Torayca cloth) to ≈30 mg cm^−2^ (1.5 mm Ni foam). Considering the loading mass of electroactive materials is less than 10 mg cm^−2^ in order to achieve reasonable performance, the conventional substrates for flexible electrodes take more than 50% inactive weight, indicating the gravimetric energy density of supercapacitor pack can be severely degraded. Graphene is a typical lightweight substrate with high conductivity and good mechanical strength, and there have been vast reports of graphene‐based flexible electrodes in the past few years, such as laser‐scribed graphene,^8^ graphene/polyaniline nanofiber,^9^ graphene–MnO_2_ composites,^10^ NiCo_2_O_4_–GO,^11^ graphene–cellulose paper,^12^ etc. However, most of earlier reports utilized graphene in oxidized form (graphene oxide), and further reduction by thermal annealing or chemical reduction is required, and there is a trade‐off between electrical conductivity and flexibility. The graphene foam (GF) is a 3D graphene material grown by chemical vapor deposition (CVD) method with open macroporous structure, inherent fast electron, high specific surface area, and light weight, which is highly desirable as lightweight, free‐standing electrode.

Typically, supercapacitors store energy through two mechanisms, which are electric double layer capacitance and pseudocapacitance, the latter one providing higher capacitance value from fast reversible faradaic reactions. More recently, metal oxides/sulfides have attracted extensive attention, as a new class of supercapacitive material, due to their high capacitance, and low cost. However, due to their poor electronic conductivity, transition metal oxides/sulfides need to be synthesized as composite nanomaterials with conductive carbon materials to achieve high electrochemical performance as pseudocapacitive materials. Transition metal oxides/hydroxides/sulfides, including Co(OH)_2_,^13^ NiO,^14^ NiCoS,^15^ CoS,^16^ etc. with versatile morphologies have been vastly investigated and high capacitance value has been reported. In efforts to develop nanostructured electrodes for electrochemical energy storage and energy conversion, the nickel or graphene foam materials are used in support of 3D scaffolds for nanomaterials, and the applications of Ni–Co–S coated on nickel foam (NF) have been reported.^17^ Graphene has a theoretical specific surface area of 2630 m^2^ g^−1^,^18^ with its conductivity closely related to the quality.^19^ The Ni–Co–S deposited on GF surface with arrayed nanostructure which is favorable for pseudocapacitive electrode materials since unlike in batteries, the redox reactions mainly occur at the electrode/electrolyte interface.

It is commonly accepted that the energy density is proportional to the square of operating voltage. Therefore, the energy density of a supercapacitor is highly dependent on the operating voltage, even more than the capacitance. The total capacitance of the device, on the other hand, is greatly limited by the electrode with the lower capacitance value, thus the design of high‐performance asymmetric supercapacitors (ASCs) requires careful selection of anode as well. There are vast reports of ASC devices using transition metal‐based cathodes, such as Co_3_O_4_(Co_3_O_4_//carbon),^20^ V_2_O_5_(V_2_O_5_/GO aerogel//V_2_O_5_/GO aerogel),^21^ and Ni–Co–S(Ni–Co–S/G//PCNS).^22^ The activated carbon with comparatively low capacitance is still applied as anodes in most cases. However, in this configuration, the capacity of the carbon material based electrode is significantly lower than its pseudocapacitive counterpart, which requires the carbon electrode to load over two times larger weight, and makes the electrode prone to cracks, reduces the mechanical strength and flexibility of the electrode. In this regard, ASC with pseudocapacitive behavior for both cathode and anode hold great promise for increasing the capacity of the supercapacitor cells. PPy is a typical p‐type conductive polymer with a broad voltage window of 1 V in alkaline electrolyte and a high capacitance value (200–550 F g^−1^) as well, which makes it promising as an anode for asymmetric supercapacitors.

We herein report the electrochemically deposited hierarchical petal‐like ternary nickel cobalt sulfides array on graphene foam, which exhibits a high capacitance of 2918 F g^−1^ at a current density of 1 A g^−1^ as the cathode of flexible ASC and PPy/GF as the anode. The flexible ASC devices have high energy densities of 79.3 and 44.7 Wh kg^−1^ at power densities of 825 and 4000 W kg^−1^, respectively.

## 2 Result and Discussion

### 2.1 Schematic Illustration of the Synthesis of the Petal‐Like Ni–Co–S

**Figure 1:**
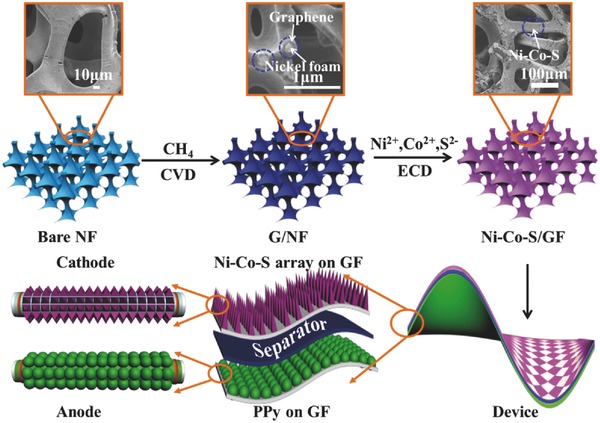
Schematic illustration of the synthesis of the petal‐like Ni–Co–S and the construction of ASC devices.

The schematic diagram of the fabrication of the petal‐like nickel cobalt sulfide nanoarrays on GF was presented in **Figure** **1**. The skeleton of the bare NF was coated with a thin graphene layer by CVD method. In order to improve its wettability, GF was treated with oxygen plasma, followed by etching with 5% HCl and 1 mol L^−1^ FeCl_3_ for 2 d to remove Ni substrate. The GF has 3D interconnected pores, which provide a high active surface area for electroactive materials deposition and can facilitate electrolyte ion trapping and reduce the electrolyte ion diffusion path during charge/discharge process. Ni–Co–S and PPy were then deposited on GF using an electrochemical method and a chemical method, respectively. ASC devices were constructed with PPy/GF and Ni–Co–S/GF as the anode and the cathode, respectively.

### 2.2 Physicochemical Characterization of the Ni–Co–S/GF

**Figure 2:**
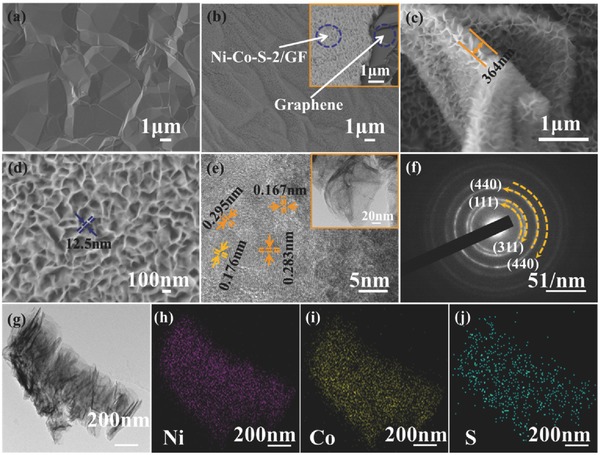
The SEM images of GF a) and Ni–Co–S‐2/GF b–d). The HRTEM image e) and the SAED pattern f) of Ni–Co–S‐2/GF. The STEM image g) of the Ni–Co–S‐2/GF and the corresponding element distribution mapping of nickel h), cobalt i), sulfur j), respectively. Inset (e) is the TEM image of Ni–Co–S‐2/GF.

The electrolyte for electrodeposition contained Ni^2+^ and Co^2+^ with the concentration of Co^2+^ fixed at 5 mmol L^−1^, while the concentration of Ni^2+^ was tuned from 5 to 10 mmol L^−1^. The thickness of Ni–Co–S nanosheet increased with smaller pore volume in‐between the Ni–Co–S interstitials when the concentration of Ni^2+^ increased from 5 to 10 mmol L^−1^ (Figure S1a–d in the Supporting Information and **Figure** **2**b). The interstitials of Ni–Co–S nanosheets reduced with the increase of the diameter of Ni–Co–S nanosheets. The structure of electrodeposited Ni–Co–S/GF sheets change from nanosheets with large interstitials (Figure S1a,b), to dense nanosheets arrays with small interstitials (Figure 2b,d), and then to even more compact nanosheet arrays (Figure S1b,d). The morphology and composition of samples have significant effects on the performance of Ni–Co–S/GF nanosheet as supercapacitor electrodes, as pseudocapacitors mainly rely on fast surface redox reactions to store charge and the surface area available for such reactions is of key importance. Figure 2c,d shows the ultrathin fluffy Ni–Co–S‐2/GF nanosheets with a thickness of ≈12.5 nm, which interconnected with each other and formed continuous Ni–Co–S‐2/GF shell layer with a thickness of ≈360–500 nm on the top of GF (Figure 2c,d). Unlike the smooth and slightly wrinkled GF, the Ni–Co–S‐2/GF nanosheet arrays were vertically aligned to the GF substrate (Figure 2a). Figure 2b shows the representative morphology of Ni–Co–S‐2/GF nanosheet, and the inset image present enlarged image of Ni–Co–S‐2/GF array on GF with boundary clearly identified. From the high‐resolution TEM (HRTEM) shown in Figure 2e, the lattice fringes of 0.283 and 0.167 nm can be ascribed to the (311) and (440) plans of CoNi_2_S (JCPDS: 24‐0334), while the 0.295 and 0.176 nm can be indexed to the (111) faces of Ni_4_S_3_ (JCPDS: 52‐1027) and the (176) faces of Co_4_S_3_ (JCPDS: 02‐1338), respectively. Representative selected area electron diffraction (SAED) pattern of Ni–Co–S‐2/GF nanosheets showed ring patterns, indicating their polycrystalline structure and each diffraction ring can also be well indexed into crystal faces of CoNi_2_S_4_ (Figure 2f). The weak X‐ray diffraction (XRD) signal intensity and diffuse ring pattern can be ascribed the small grain size of Ni–Co–S (Figure S4, Supporting Information). The energy dispersive spectroscopy (EDS; Figure S3, Supporting Information) shows that roughly the same amounts of Ni and Co are present in the sample, indicating the existence of Co_4_S_3_ in the sample. The intense C signal mainly comes from graphene foam, and the O signal comes from oxide and hydroxide impurities, adsorbed adventitious species, and gas in the chamber. The S signal is weaker than anticipated from the stoichiometry of sulfides mainly due to the thickness of the sample being much smaller than the interaction volume, resulting in a large amount of signal loss for light elements such as S. The EDS mapping of Ni, Co, and S elements in Ni–Co–S/GF are shown in Figure 2g–j, with all the elements distributed uniformly in Ni–Co–S‐2/GF nanosheet arrays, indicating the formation of metal sulfides. The one‐step electrodeposition is an effective approach to achieve hierarchical structured ternary transition metal sulfides with uniform nanosheet arrays and is advantageous to other multistep chemical fabrication techniques followed by ion‐exchange.^23^ The unique fluffy petal‐like Ni–Co–S/GF interconnected to form continuous honeycomb‐like Ni–Co–S layers on highly conductive GF with a large amount of interstitial pores which facilitate ion diffusion and provide large electrode/electrolyte interface for pseudocapacitive charge generation.

**Figure 3:**
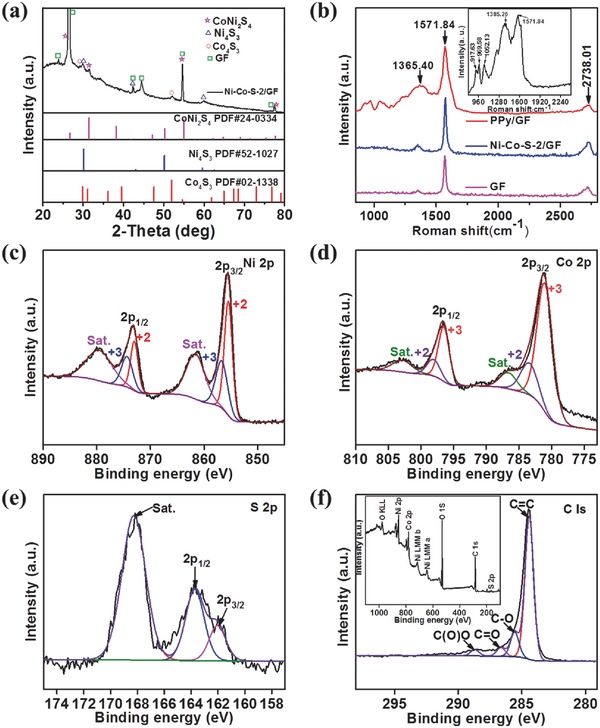
The XRD patterns a) of Ni–Co–S/GF and GF. Raman spectra b) obtained for PPy/GF, Ni–Co–S/GF, GF, and PPy (inset figure (b)). XPS spectrum of Ni–Co–S/GF: c) Ni 2p, d) Co 2p, e) S 2p, (f) C 1s and inset figure (f) is XPS survey spectrum of Ni–Co–S/GF.

The Ni–Co–S‐2/GF prepared by the electrochemical method is highly crystalline (**Figure** **3**a), with diffraction peaks at 31.47° and 55.01° can be indexed to the (311) and (440) planes of CoNi_2_S_4_ (JCPDS: PDF#24‐0334), respectively. The strong diffraction peak intensity at 30.14° and 51.91° can be ascribed to the (111) and (440) planes of Ni_4_S_3_ (JCPDS: PDF#52‐1027) and Co_4_S_3_ (JCPDS: PDF#02‐1338), respectively. Compared to the XRD spectra of Ni–Co–S‐1/GF (Figure S4a, Supporting Information) and Ni–Co–S‐3/GF (Figure S4b, Supporting Information), the intensity of peaks associated the redox active and electrical conductive CoNi_2_S_4_ was stronger, which indicated the better performance of Ni–Co–S‐2/GF as a supercapacitor electrode. Moreover, it is generally regarded to be very difficult to achieve high‐quality XRD result by electrodeposition method.^24^ There was no significant difference between the samples of Ni–Co–S‐1/GF, Ni–Co–S‐2/GF, and Ni–Co–S‐3/GF with the increasing of the Ni^2+^ concentration, indicating the change of the concentration of Ni^2+^ cannot lead to the change of crystal structure, may be only the proportion of matter produced. The Raman spectra of PPy/GF, Ni–Co–S‐2/GF, GF, and pure PPy (inset of Figure 3b) are shown in Figure 3b. The GF has typical D band at 1365.40 cm^−1^, G band at 1571.84 cm^−1^, and 2D band at 2738.01 cm^−1^, which can be ascribed to the structural disorder, the E_2g_ vibration mode of graphite‐type sp^2^ carbons, and the number of layers of the GF, respectively.^25^ The *I*_G_ to *I*_D_ ratio shows both the degree of graphitization and amount of defects on the graphene foam.^26^ The Ni–Co–S‐2/GF has higher *I*_D_/*I*_G_ value which indicates the generation of defects after O_2_ plasma treatment or electrochemical deposition; however, the majority of the sp^2^ structure was well maintained in the composites. Overall the GF has high conductivity, a large amount of sp^2^‐hybridized carbon components, and few defects, which is different from the conventionally used rG‐O/G‐O^27^ prepared by the chemical method which has a lot of defects even after reduction. The PPy/GF has resonance peaks at 917.63 and 969.58 cm^−1^ which can be ascribed to the N—H and C—H deformation of the pyrrole ring, while the peaks at 1385.26 and 1571.84 cm^−1^ can be assigned to C=C stretching of PPy. Raman spectroscopy confirms the deposition of PPy on GF. XPS measurement was performed on the Ni–Co–S‐2/GF to analyze the surface elemental composition and chemical valence states of different elements. The survey spectrum (inset Figure 3) shows that Ni–Co–S‐2/GF consists of Ni, Co, S, O, and C, in which the element O comes from oxygen plasma pre‐treatment of graphene foam, adventitious moisture, and oxygen‐containing groups resulted from exposure in air and the element C is from both GF exposed on the surface and the reference carbon. Therefore, Ni, Co, and S are the main chemical compositions in the near‐surface of Ni–Co–S/GF samples. Figure 3c–f shows the typical Ni 2p, Co 2p, S 2p, and C 1s narrow scan of the Ni–Co–S‐2/GF sample. The Ni 2p and Co 2p spectra of XPS can be well fitted with two spin–orbit splitting which is characteristic of Ni^2+^/Ni^3+^, Co^2+^/Co^3+^, respectively, and two shakeup satellites (denoted as “Sat.”). As shown in Figure 3c, the binding energies of Ni 2p peaks located at 855.58 and 873.18 eV correspond to Ni 2p_3/2_ and Ni 2p_1/2_, respectively. The binding energies of Ni 2p peaks located at 855.48 and 872.98 eV indicate the existence of Ni^2+^, which is probably due to impurities containing Ni^2+^, while the binding energies at 856.78 and 874.38 eV can be assigned to Ni^3+^, which comes from the samples of Ni_4_S_3_ and CoNi_2_S_4_.^28^ Similarly, for the Co 2p XPS spectrum in Figure 3d, the peaks at 781.18 and 796.68 eV are related to Co 2p_3/2_ and Co 2p_1/2_, respectively. The spin–orbit doublets situated at 783.58 and 798.18 eV can be ascribed to Co^2+^, which forms the CoNi_2_S_4_ sample, while binding energies at 781.08 and 796.85 eV correspond to the spin–orbit splitting of Co^3+^. The intensity of the peaks indicates that Co^3+^ is the dominant state of Co near the surface of the sample and comes from the Co_4_S_3_ sample, which is consistent with the XRD results. The weak satellite peaks indicate Co^3+^ was the main presence state for the Co.^29^ The S 2p narrow scan is shown in Figure 3e, which can be divided to two peaks at 163.68 and 162.08 eV correspond to S 2p_1/2_ and S 2p_3/2_, respectively. For the C1s XPS spectrum hybrids in Figure 3f, the spectrum can be fitted and divided into four types of carbon bonds: the aromatic‐linked carbon (C=C, 284.38 eV); the C in oxygen single‐bonded carbon bonds (C—O, 285.58 eV); the carbonyl carbon (C=O, 286.68 eV), and the carboxylate carbon (O—C=O, 288.68 eV).^30^ This indicates that some oxygenated groups are still present on the graphene foam surface under the electrodeposition conditions. Moreover, these oxygenated groups are beneficial for intimate anchoring Ni–Co–S array on graphene foam and enhancing the structural stability of the electrodes. According to the XPS analysis, the near‐surface of the Ni–Co–S sample contains Ni^2+^, Ni^3+^, Co^2+^, Co^3+^, S^2−^, and S^4−^. These results are in good agreement with earlier reports of the chemical states of Ni and Co binary metal sulfides.^31^

### 2.3 Electrochemical Characterization of the Ni–Co–S/GF and PPy/GF

**Figure 4:**
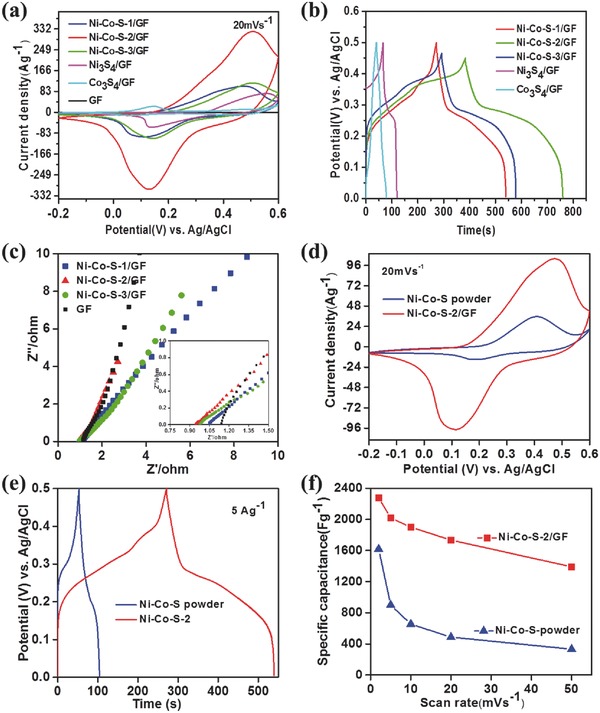
Electrochemical performance of the Ni–Co–S nanosheets as supercapacitor electrodes in the three‐electrode measurements with 1 mol L^−1^ KOH as the electrolyte. a) Cyclic voltammetry curves, b) galvanostatic charge/discharge curves, and c) Nyquist impedance spectra of the Ni–Co–S nanosheets with different concentration of Ni^2+^. d) Cyclic voltammetry curves, e) galvanostatic charge/discharge curves, and f) the gravimetric specific capacitance values of the optimized Ni–Co–S‐2/GF in comparison with chemically synthesized Ni–Co–S powder.

The electrochemical performances of Ni–Co–S nanosheet arrays prepared from different Ni^2+^ ratios were systematically investigated as shown in **Figure** **4**a. The Ni–Co–S/GF prepared from 5 mmol L^−1^ Co^2+^ with various amounts (5, 7.5, and 10 mmol L^−1^) of Ni^2+^, were denoted as Ni–Co–S‐1/GF, Ni–Co–S‐2/GF and Ni–Co–S‐3/GF, respectively. The Ni_3_S_4_/GF and Co_3_S_4_/ GF were obtained with the same electrodeposition method of Ni–Co–S/GF. The anodic peaks in the cyclic voltammetry (CV) curves shift to higher potential with the increase of Ni^2+^ concentration, which agrees with the electrochemical behaviors of cobalt sulfides and nickel sulfides reported previously.^32^ The Ni–Co–S‐2/GF sample has the smallest anodic and cathodic peaks separation in the batch, indicating its high reversibility for redox reactions. Furthermore, the specific capacitance of Ni–Co–S electrodes varied with increasing of Ni^2+^ concentration due to their different elemental composition and surface structural features. For example, the specific capacitance of Ni–Co–S‐2/GF at 10 A g^−1^ was 2550 F g^−1^, significantly higher than that of 893.33 F g^−1^ for Ni–Co–S‐1/GF and 956.67 F g^−1^ of Ni–Co–S‐3/GF (Figure 4b). Ni–Co–S‐2/GF prepared by the electrochemical deposition in CV technique with 7.5 mmol L^−1^ Ni^2+^ and 5 mmol L^−1^ Co^2+^ for 15 cycles has the highest electrochemical capacitance. The resulting electrochemical performance is comparable or superior to that of most of the nanostructured Ni–Co–S materials reported previously (see **Table** **1** for a detailed comparison).

**Table 1:** Electrochemical properties for Ni–Co–S/GF of this work in comparison with those Ni‐ and/or Co‐based transition metal sulfides in earlier reports

Ni–Co–S‐based electrode	Voltage range	Specific capacitance	Rate capability	Ref.
Petal‐like Ni–Co–S nanosheet	−0.2V to 0.6 V (vs Ag/AgCl)	405.27 mAh g^−1^ at 1 A g^−1^	328.33 mAh g^−1^ (81.02%) at 1 A g^−1^	This work
Carbon@NiCo_2_S_4_ nanorods	0–0.45 V (vs Ag/ AgCl)	182 mAh g^−1^ at 1 A g^−1^	158 mAh g^−1^ (86.7%) at 10 A g^−1^	47
Ni–Co–S nanosheet arrays	−0.2 to 0.6 V (vs Ag/ AgCl)	197 mAh g^−1^ at 5 A g^−1^	178 mAh g^−1^ (90.6%) at 100 A g^−1^	34
CoNi_2_S_4_ nanosheet arrays	0–0.45 V (vs SCE)	363 mAh g^−1^ at 5 mA cm^−2^	284 mAh g^−1^ (78.1%) at 50 mA cm^−2^	39
NiCo_2_S_4_ mesoporous nanosheets	0–0.5 V (vs Hg/HgO)	103 mAh g^−1^ at 1 A g^−1^	86 mAh g^−1^ (83.3%) at 20 A g^−1^	48
NiCo_2_S_4_ flaky arrays	−0.1 to 0.5 V (vs SCE)	284 mAh g^−1^ at 1 A g^−1^	145 mAh g^−1^ (51.1%) at 8 A g^−1^	49
Core–shell NiCo_2_S_4_ nanostructures	0–0.5 V (vs Hg/HgO)	271 mAh g^−1^ at 1 mA cm^−2^	215 mAh g^−1^ (79.4%) at 20 mA cm^−2^	50
NiCo_2_S_4_ nanoparticles	−0.2 to 0.4 V (vs Ag/ AgCl)	190 mAh g^−1^ at 1 A g^−1^	129 mAh g^−1^ (67.9%) at 40 A g^−1^	51
Hollow Ni*_x_*Co_9−_ *_x_*S_8_ @N‐doped carbon	0–0.45 V (vs Ag/ AgCl)	176 mAh g^−1^ at 2 A g^−1^	73 mAh g^−1^ (41.3%) at 8 A g^−1^	52
NiCo_2_S_4_ nanotube arrays	0–0.55 V (vs Hg/HgO)	366 mAh g^−1^ at 5 mA cm^−2^	248 mAh g^−1^ (67.7%) at 150 mA cm^−2^	34
NiCo_2_S_4_ porous nanotubes	−0.1 to 0.5 V (vs Hg/HgO)	152 mAh g^−1^ at 0.2 A g^−1^	76 mAh g^−1^ (50.3%) at 5 A g^−1^	32
Ni*_x_*Co_3−_ *_x_*S_4_ hollow nanoprisms	0–0.5 V (vs SCE)	124 mAh g^−1^ at 1 A g^−1^	81 mAh g^−1^ (65.4%) at 20 A g^−1^	38
Ni–Co sulfide nanowires	0–0.45 V (vs Ag/ AgCl)	302 mAh g^−1^ at 2.5 mA cm^−2^	147 mAh g^−1^ (48.7%) at 30 mA cm^−2^	53
CoNi_2_S_4_/graphene nanocomposite	0–0.38 V (vs SCE)	212 mAh g^−1^ at 1 A g^−1^	110 mAh g^−1^ (52.1%) at 20 A g^−1^	33
Carbon‐NiCo_2_S_4_ nanosheet arrays	−0.2 to 0.8 V (vs SCE)	368 mAh g^−1^ at 2 mA cm^−2^	146 mAh g^−1^ (39.6%) at 200 mA cm^−2^	54
Ni–Co–S ball‐in‐ball hollow spheres	−0.1 to 0.55 V (vs SCE)	158 mAh g^−1^ at 1 A g^−1^	108 mAh g^−1^ (68.1%) at 20 A g^−1^	55
Urchin‐like NiCo_2_S_4_	0–0.565 V (vs Hg/HgO)	180 mAh g^−1^ at 1 A g^−1^	139 mAh g^−1^ (77.3%) at 20 A g^−1^	56
CNTs@Ni–Co–S nanosheet core/shell arrays	−0.2 to 0.6 V (vs SCE)	222 mAh g^−1^ at 4 A g^−1^	193 mAh g^−1^ (87.1%) at 50 A g^−1^	57
NiCo_2_S_4_ nanosheets on graphene	0–0.5 V (vs Ag/ AgCl)	202 mAh g^−1^ at 3 A g^−1^	106 mAh g^−1^ (52.4%) at 20 A g^−1^	58

To further evaluate the ion‐diffusion of the electrodes, electrochemical impedance spectroscopy (EIS) measurements were conducted with frequency from 100 MHz to 100 kHz. The intercept with the *x* axis at high frequency indicates the series resistance (*R*_s_), which is the sum of electrolyte resistance, the contact resistance of the electrode materials with current collector, and electrolyte. The Nyquist plot of Ni–Co–S/GF (Figure 4c) contains one semicircle at high‐frequency region and goes linearly at low frequency, which corresponds to the charge transfer resistance and semi‐diffusion process, respectively. The Ni–Co–S/GF has low contact resistance and capacitive behavior with low diffusion resistance in low‐frequency region. Firstly, the ultrathin nanosheet arrays of Ni–Co–S layers offered a high electrode/electrolyte interface, with numerous electroactive surface sites for redox reactions. Secondly, the interstitials of these ultrathin vertically aligned nanosheet arrays could sever as a reservoir for the electrolyte, and significantly enhance the diffusion of the electrolyte ions. Thirdly, the GF with high electrical conductivity serves as an ideal substrate for the strong attachment of Ni–Co–S nanosheets, which also has a fast transportation of pseudocapacitive charge. The electrochemically synthesized Ni–Co–S has a larger area of CV curve than that of Ni–Co–S powder at a scan rate of 20 mV s^−1^ (Figure 4d). Galvanostatic charge/discharge (GCD) profiles of Ni–Co–S with different Ni^2+^ concentration, compared with the Ni–Co–S powder at a current density of 5 A g^−1^ are shown in Figure 4b,e. GCD curves show typical supercapacitive characteristics with very small voltage drops in the discharge process, indicating good electric and ionic conductivity of the ternary transition metal sulfides. Moreover, the capacitance retention of Ni–Co–S‐2/GF at a scan rate of 50 mV s^−1^ is 61.04% compared to that at 2 mV s^−1^ (Figure 4f), superior to that of the Ni–Co–S powder, which is 20.57%. The Ni–Co–S synthesized by electrochemical deposition method not only has higher capacitance value but also better rate performance than that of chemically synthesized Ni–Co–S powder, which can be attributed to the superior charge storage behavior of vertically aligned ultrathin nanosheets structure for Ni–Co–S‐2/GF than the dense agglomerates structure of the Ni–Co–S powder. The Ni–Co–S‐2/GF electrodes were selected as the cathode for further studies, and denoted as Ni–Co–S/GF, except further mentioned.

**Figure 5:**
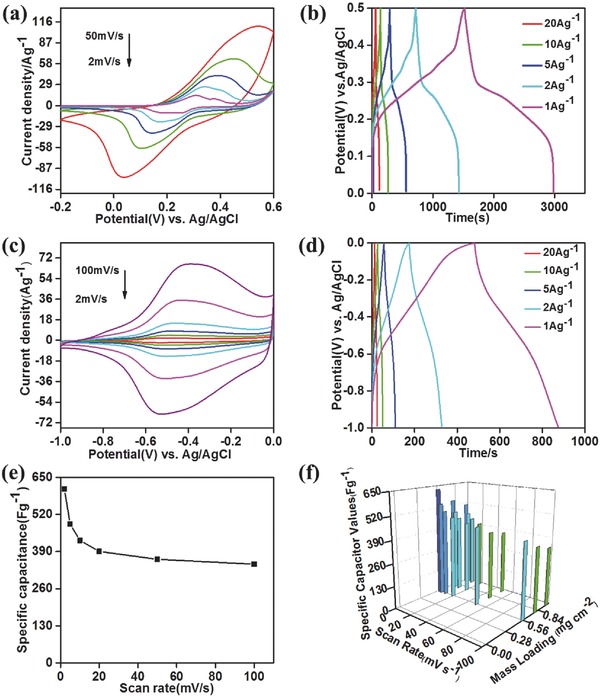
Electrochemical characterization of Ni–Co–S/GF and PPy/GF electrodes. CV curves of Ni–Co–S/GF a) and PPy/GF c) at various scan rates from 2 to 100 mV s^−1^. Galvanostatic charge/discharge curves of Ni–Co–S/GF b) and PPy/GF e) at different current densities from 1 to 20 A g^−1^. Comparison of the specific capacitance values as a function of current densities for PPy/GF e). The gravimetric specific capacitance values of PPy/GF with different mass loadings of PPy f).

The electrochemical performance of the Ni–Co–S/GF nanosheets as the cathode was investigated systematically in the three‐electrode system with 1 mol L^−1^ KOH as the electrolyte. The CVs curves of Ni–Co–S/GF at different scan rates show a pair of redox peaks as shown in **Figure** **5**a, representing a typical electrochemical behavior of the Ni‐ or Co‐based transition metal sulfide electrodes.[[qv: 32a,33]] The existence of the redox peaks can be attributed to the reactions between the alkaline electrolyte and the electrode material through the following equation (1), 34

(1) $${\mathrm{CoNi}}_{2}S_{4}+2{\mathrm{OH}}^{-}↔{\mathrm{CoS}}_{2x}\mathrm{OH}+{\mathrm{Ni}}_{2}S_{4–2x}\mathrm{OH}+{\mathrm{2e}}^{-}$$

The reversible redox reactions lead to the formation of Ni_2_S_4−2_ *_x_*OH and CoS_2_ *_x_*OH, similar to the reaction mechanism of NiCo*_x_*O*_y_* reported previously.^35^ From the Figure 5a, The Ni–Co–S/GF electrode gave rise to an anodic and a cathodic peak at 0.4 and 0.14 V, respectively, corresponding to the redox reactions of Ni–Co–S/GF. In addition, the OH^−^ ions play a significant role in the electrochemical oxidation and reduction of nickel sulfides and cobalt sulfides as reported previously.^36^ Rate capability is an important parameter estimating the power density of electrochemical capacitors. Figure 5b shows the representative GCD curves of Ni–Co–S/GF nanosheets electrode at the current densities of 1, 2, 5, 10, and 20 A g^−1^. The plateaus in the charge/discharge curves indicate the pseudocapacitive behavior of transition metal sulfides due to the reversible redox reactions of Ni–Co–S/GF. GF which has an areal density of 5 mg cm^−2^ was deposited with PPy at an areal density of 3.7 mg cm^−2^ by a chemical method, with synthetic procedures shown in experiment section. The CV curves of PPy/GF at different scan rates from 2 to 100 mV s^−1^ show obvious oxidation and reduction peaks of PPy at −0.39 and −0.55 V, respectively (Figure 5c), which demonstrate the highly reversible redox reaction. The GCD curves of PPy/G at different current densities are shown in Figure 5d, the capacitance of PPy/G are 420, 322, 273, 249, and 236 F g^−1^, at current densities of 1, 2, 5, 10, and 20 A g^−1^, respectively. The increase of the mass loading of the active materials is important to achieve high gravimetric capacitance. The electrochemical performance of PPy/G with different mass loadings of PPy was investigated, as shown in Figure 5f. The gravimetric specific capacitance increases from 424 to 478 F g^−1^ when the loading of PPy decreases from 1.09 to 0.56 mg, at a scan rate of 10 mV s^−1^.

### 2.4 Electrochemical Characterization of the Asymmetric Supercapacitors

**Figure 6:**
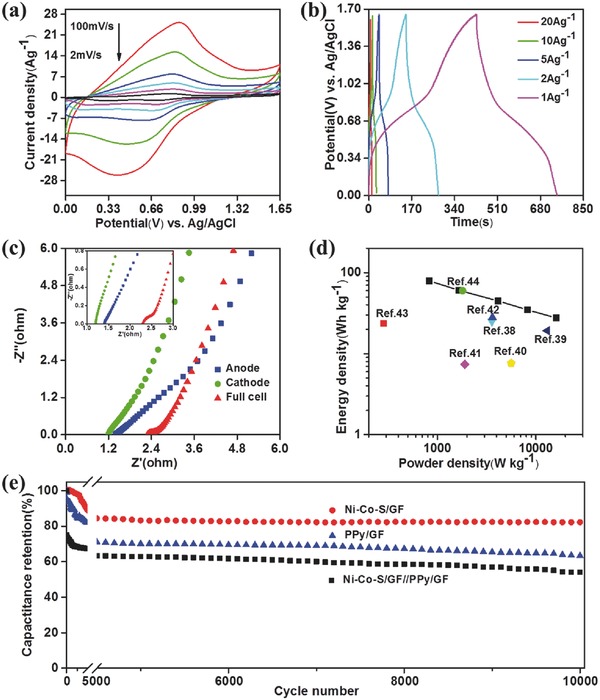
Electrochemical characterization of Ni–Co–S/GF//PPy/GF‐based ASC device. CV curves a) at different scan rates, galvanostatic charge/discharge curves b) at different current densities from 1 to 20 A g^−1^ and energy and power densities d) of ASC devices. Nyquist plots c) of the anode, and cathode, and the ASC cell. e) Cycling performance of PPy/GF, Ni–Co–S/GF and the as‐assembled ASC devices up to 10 000 cycles.

ASC devices were assembled with Ni–Co–S/GF as the cathode and PPy/GF as the anode in 1 M KOH electrolyte. The CV curves of the supercapacitor devices (**Figure** **6**a) have a pair of redox peaks at various scan rates from 2 to 100 mV s^−1^, which can be attributed to the Faradic charge of Ni–Co–S/GF. GCD curves of the supercapacitors at various current densities from 1 to 20 A g^−1^ are illustrated in Figure 6b. The specific capacitance of the supercapacitors calculated from the GCD curve is 209.82, 118.2, and 93.0 F g^−1^ at current densities of 1, 5, and 10 A g^−1^, respectively. The ASC device capacitance is calculated based on the total mass of the two electrodes, including PPy and the active materials. The Nyquist plots (Figure 6c) show that the charge‐transfer resistance values are 1.2, 1.4, and 2.3 ohms for the cathode, anode, and ASC device, respectively. Power density and energy density are two critical parameters to evaluate the capacitive performance for a two‐electrode supercapacitor device. The energy and power densities were calculated according to the following equations^37^

(2) $$E=\frac{CV^{2}}{2\times 3.6}$$

(3) $$P_{\mathrm{real}}=\frac{E}{t}$$

where *C* in F g^−1^ is the capacitance value of the supercapacitor cell, *m* is the total mass of electroactive materials, including PPy and Ni–Co–S/GF at both electrodes. Figure 6d shows the Ragone plot relating the energy density to the power density of ASC devices. Notably, the energy density of ASC devices is comparable or superior than that of Ni‐Co sulfide nanowire//activated carbon cells (25 Wh kg^−1^ at 3.57 kW kg^−1^),^38^ carbon/CoNi_3_O_4_//activated carbon cells (19.2 Wh kg^−1^ at 13 kW kg^−1^),^39^ graphene–nickel cobaltite nanocomposite//activated carbon (7.6 Wh kg^−1^ at 5.6 kW kg^−1^),^40^ Ni–Co oxide//activated carbon (7.4 Wh kg^−1^ at 1.9 kW kg^−1^),^41^ Co*_x_*Ni_1−_ *_x_*O/reduced G–O//reduced G–O cells (28 Wh kg^−1^ at 3614 W kg^−1^),^42^ Ni–Co hydroxides/Zn_2_SnO_4_//activated carbon (AC) devices (23.7 Wh kg^−1^ at 284 W kg^−1^),^43^ Ni–Co–S/cloth//GF (60 Wh kg^−1^ at 1.8 kW kg^−1^),^44^ and CoNi_2_S_4_ nanosheet arrays on NF//AC devices (33.9 Wh kg^−1^ at 409 W kg^−1^).^45^ The electrochemical properties of nickel cobalt sulfide‐based ASCs are generalized in Table S2 (Supporting Information). The energy density of ASC device is 79.3 Wh kg^−1^ at 825 W kg^−1^, superior to that of aqueous and organic electrolyte based electric double‐layer capacitors (EDLCs)(3–15 Wh kg^−1^). As a crucial parameter determining the practical applications of supercapacitors, long term cycle life of the ASC devices was evaluated by GCD method at a current density of 5 A g^−1^ for 10 000 cycles. The capacitance retention for ASC devices is 54.02%, while the capacitance retention of Ni–Co–S/GF and PPy/GF are 82.26% and 63.37%, respectively after 10 000 cycles. In order to match the areal capacitance of PPy/GF with that of Ni–Co–S/GF, the PPy mass loading should be higher than 5 mg cm^−2^. The fairly high loading of PPy may be the main reason resulting in the poor cyclicity of the PPy/GF (63.37% of capacity retention). The ASC device maintained 54.02% of the initial capacity after 10 000 cycles. Also, the manual assembly process in the laboratory may lead to the capacitance degradation with cycling. Nevertheless, the capacitance of the device after 10 000 cycles can be still maintained above 140 F g^−1^, which is higher than that of AC‐based devices tested in our lab.

### 2.5 Performance of the Asymmetric Supercapacitors

**Figure 7:**
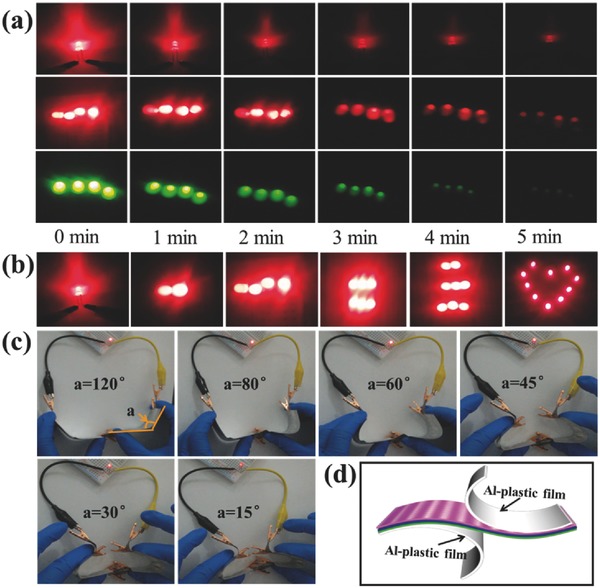
a) The time of the LEDs lighting with different number and lowest working voltage was powered by two of our ASCs in series. b) Pictures of two ASCs powering 1, 2, 4, 6, 8, and 10 LEDs, respectively. c) Photograph of flexible ASC working under different bending angle from 15° to 120° and d) schematic diagram of flexible device.

Numerous materials such as metal foam, metal foil, and carbon cloth have been used as substrates for binder‐free, flexible supercapacitor electrodes, but they take more than 40% weight of the electrode, indicating the gravimetric energy density in supercapacitor pack may be severely degraded. GF with areal density smaller than 3 mg cm^−2^, which is 100 times smaller than that of Ni foam (≈30 mg cm^−2^, 1.5 mm thick), indicating their potential for flexible supercapacitor electrodes, and their applicability as flexible energy storage devices has been demonstrated as below. Two of 1 cm^2^ ASC devices were connected in series to light up nine light‐emitting diode (LED) lights, which can operate for around 7 min after charging for 150 s (at 1 A g^−1^), as shown in **Figure** **7**. Four green LEDs with lowest working voltage of 3.2 V can also be lit up with two of 1 cm^2^ ASC connected in series, indicating the high operating potential of ASC devices at 1.6 V. To evaluate the mechanical flexibility of the ASC cell, the ASC cells were bent to different angles while lightening the LEDs, or under CV test. No apparent changes in the brightness of LEDs lighten by the ASC devices, or CV curves shape were observed under different bending angles from 0 to 180°. The video in Supporting Information showed ten of LEDs lightened by two ASC devices at various bending angles and durations. These results further proved the excellent flexibility and electrochemical stability of the device under flexed conditions, which can be ascribed to the synergistic effect of hierarchical structure of vertically aligned Ni–Co–S nanosheet arrays and highly conductive GF substrate. By virtue of superior specific capacitance, high energy/power densities and mechanically robustness, the electrochemical deposition of Ni–Co–S providing a new strategy to fabricate hierarchically structured transition metal sulfides on porous substrates for flexible and high‐performance energy related applications.

## 3 Conclusion

In summary, Ni–Co–S nanosheet arrays on 3D porous graphene foam have been successfully synthesized by a single‐step electrochemical deposition process, and a robust, flexible supercapacitor device with a Ni–Co–S/GF cathode and a PPy/GF anode has been demonstrated with electrochemical performance systematically evaluated. The concentration of Ni^2+^ precursor plays an important role in determining the structural features and has pronounced effect on the electrochemical performance of Ni–Co–S/GF. The 3D hierarchically structured Ni–Co–S/GF synthesized from 0.75 mmol L^−1^ of NiCl_2_·6H_2_O has the highest specific capacitance of 2918 and 2364 F g^−1^ at a current density of 1 and 20 A g^−1^, respectively. Flexible ASC devices have been assembled with a high full cell potential of 1.65 V, which delivers an excellently high energy density values of 79.3 and 37.7 Wh kg^−1^ at power densities of 825.0 and 16100 W kg^−1^, respectively. No obvious changes of the as‐measured CV curves and the brightness of LED were observed when the cells were bent from 15° to 180°. The free‐standing feature and excellent flexibility open much more opportunities for a wide range of applications. From the above, we believe that the electrochemical synthesis of Ni–Co–S on 3D porous structure is an efficient strategy to construct free‐standing and flexible electrodes, the Ni–Co–S/GF//PPy/GF supercapacitor devices has impressive properties which can be expected to serve as a promising platform for the development of flexible energy storage devices.

## 4 Experimental Section

*Reagents and Materials*: All chemicals used in this study were received from Sinopharm Chemical Reagent Co. Ltd and used without further treatment.

*Synthesis of Graphene/Nickel Foam*: The preparation of graphene foam followed a procedure similar to that reported in the previous study.^46^ Typically, a nickel foam with a pore density of 100 pores per inch and thickness of 1 mm was treated and used as the template for growth of 3D graphene foam. The template with dimensions of 10 cm × 5 cm was firstly washed carefully with ethanol and was then placed inside a horizontal quarts tube in a First NanoET3000 CVD system. In order to remove the atmospheric oxygen and water on the surface of the nickel foam, the tube was pumped down to 100 mTorr, and then a gas mixture of Ar and H_2_ with the rate of 10:1 was filled into the tube. The tube was heated to 1000 °C under a gas flow of Ar (500 sccm) and H_2_ (100 sccm), and kept at 1000 °C for 10 min to clean the impurities on the surface of nickel. After that a gas flow of CH_4_ (100 sccm) was injected into the chamber while increasing Ar flow to 800 sccm and keeping the same H_2_ flow rate. After maintaining the reaction environment for 10 min, the gases were purged and the chamber was cooled down to room temperature. To increase the wettability of the graphene/nickel foam, it was treated with oxygen plasma with a power of 80 W for 30 s.

*Synthesis of Ni–Co–S/Graphene Foam (Ni–Co–S/GF)*: The graphene/nickel foam was washed in solution of 5% HCl and 1 mol L^−1^ FeCl_3_ for 2 d to remove nickel substrate. The nickel cobalt sulfide (Ni–Co–S) was electrodeposited on the graphene foam.^44^ The graphene foam was wetted and cleaned with acetone, ethanol followed by deionized water. The electrodeposition solution contained 5 mmol L^−1^ CoCl_2_·6H_2_O, 0.075 mol L^−1^ thiourea (CS(NH_2_)_2_), and different concentrations of 5, 7.5, and 10 mmol L^−1^ NiCl_2_·6H_2_O were denoted as Ni–Co–S‐1/GF, Ni–Co–S‐2/GF, and Ni–Co–S‐3/GF, respectively. The pH value of the solution was adjusted to ≈6 with diluted ammonic solution. Cyclic voltammetry was applied at a scan rate of 5 mV s^−1^ for 15 cycles within a potential range from −1.2 to 0.2 V versus Ag/AgCl. The electrodeposited graphene foam was cleaned by rinsing with a large amount of water, followed by drying in air for 6 h, and vacuum drying at 80 °C for 6 h. The typical mass loading of the Ni–Co–S/GF cathode was 1 mg cm^−2^.

*Synthesis of PPy/GF*: The graphene foam was weighed and put in a beaker containing 100 mL of 0.1 mol L^−1^ HCl and 0.1 mmol (6.9 mL) of pyrrole. The beaker was then put into an ice bath and magnetically stirred for 10 min to allow the substrate to fully absorb the solution. 3 mL of 0.1 mol L^−1^ FeCl_3_ and 0.1 mol L^−1^ LiClO_4_ solutions were added drop by drop into the mixture. The reaction was then kept for 8 h. The product was then washed with ethanol and DI water, and dried at 60 °C under vacuum.

*Fabrication of Ni–Co–S/GF//PPy/GF Asymmetric Supercapacitors*: The Ni–Co–S/GF//PPy/G asymmetric supercapacitors were fabrication by using the Ni–Co–S/GF nanosheets and PPy/ as cathode and anode, respectively. 1 mol L^−1^ KOH solution was used as the electrolyte, and a porous filter paper was selected as the separator (GB/T1914‐2007). Before the fabrication of the asymmetric supercapacitor, the masses of the cathode and anode were balanced according to the following equation (4)

(4) $$\frac{m_{+}}{m_{-}}=\frac{C_{S-}\Delta V_{-}}{C_{S+}\Delta V_{+}}$$

where *m* is the mass, *C*_S_ is the specific capacitance, and Δ*V* is the voltage range for cathode (+) and anode (−), respectively. The typical mass loading of an ASC is about 1.5 mg.

*Materials characterization*: The structures, morphologies, and chemical compositions of the samples were characterized by scanning electron microscopy (SEM; JEOL JSM‐7800F). TEM (JEOL‐2100) and high‐resolution transmission electron microscopy (HRTEM; JEOL JEM‐2010F), Raman spectra were obtained via a confocal microscope Raman spectrometer system (Witec Alpha 300; with the wavelength excitation laser of 488 nm) and XRD (Bruker Focus D8 with Cu Kα radiation, Smart lab).

*Electrochemical Measurements*: The electrochemical measurements were carried out at room temperature in both two‐electrode and three‐electrode system. In the three‐electrode measurements, Ni–Co–S/GF was used as the working electrode, a Pt piece as the counter electrode, and Ag/AgCl as the reference electrode. In the full‐cell measurement, an ASC was assembled with Ni–Co–S/GF acting as cathode and PPy/GF as anode. The electrolyte for all electrochemical measurements was 1 mol L^−1^ KOH solution. The electrochemical property was tested by CHI760E by the techniques of CV, GCD, and EIS. The voltage window for the Ni–Co–S/GF cathode, the PPy/GF anode, and the asymmetric Ni–Co–S/GF//PPy/GF cell was from −0.2 to 0.6 V, −1 to 0 V and 0 to 1.65 V versus Ag/AgCl, respectively.

## Conflict of Interest

The authors declare no conflict of interest.

## Supporting information

Supplementary

Supplementary

Supplementary
