# Supplementary material for: Electrochemically Synthesis of Nickel Cobalt Sulfide for High‐Performance Flexible Asymmetric Supercapacitors
Source: Adv Sci (Weinh). 2017 Dec 2;5(2):1700375. doi: 10.1002/advs.201700375 (PMC5827014; doi:10.1002/advs.201700375)
Supplement: Supplementary file 1 — Supplementary [file ADVS-5-1700375-s002.pdf]

Copyright WILEY-VCH Verlag GmbH & Co. KGaA, 69469 Weinheim, Germany, 2017.

ADVANCED SCIENCE
Open Access

# Supporting Information

for *Adv. Sci.,* DOI: 10.1002/advs.201700375

# Electrochemically Synthesis of Nickel Cobalt Sulfide for High-Performance Flexible Asymmetric Supercapacitors

*Chunyan Zhang, Xiaoyi Cai, Yao Qian, Haifeng Jiang, Lijun Zhou, Baosheng Li, Linfei Lai,* Zexiang Shen, and Wei Huang*

Supporting Information

**Electrochemically Synthesis of Nickel Cobalt Sulfide for High-Performance Flexible Asymmetric Supercapacitors**

*Chunyan Zhang, Xiaoyi Cai, Yao Qian, Haifeng Jiang, Lijun Zhou, Baosheng Li, Linfei Lai,* Zexiang Shen, and Wei Huang**

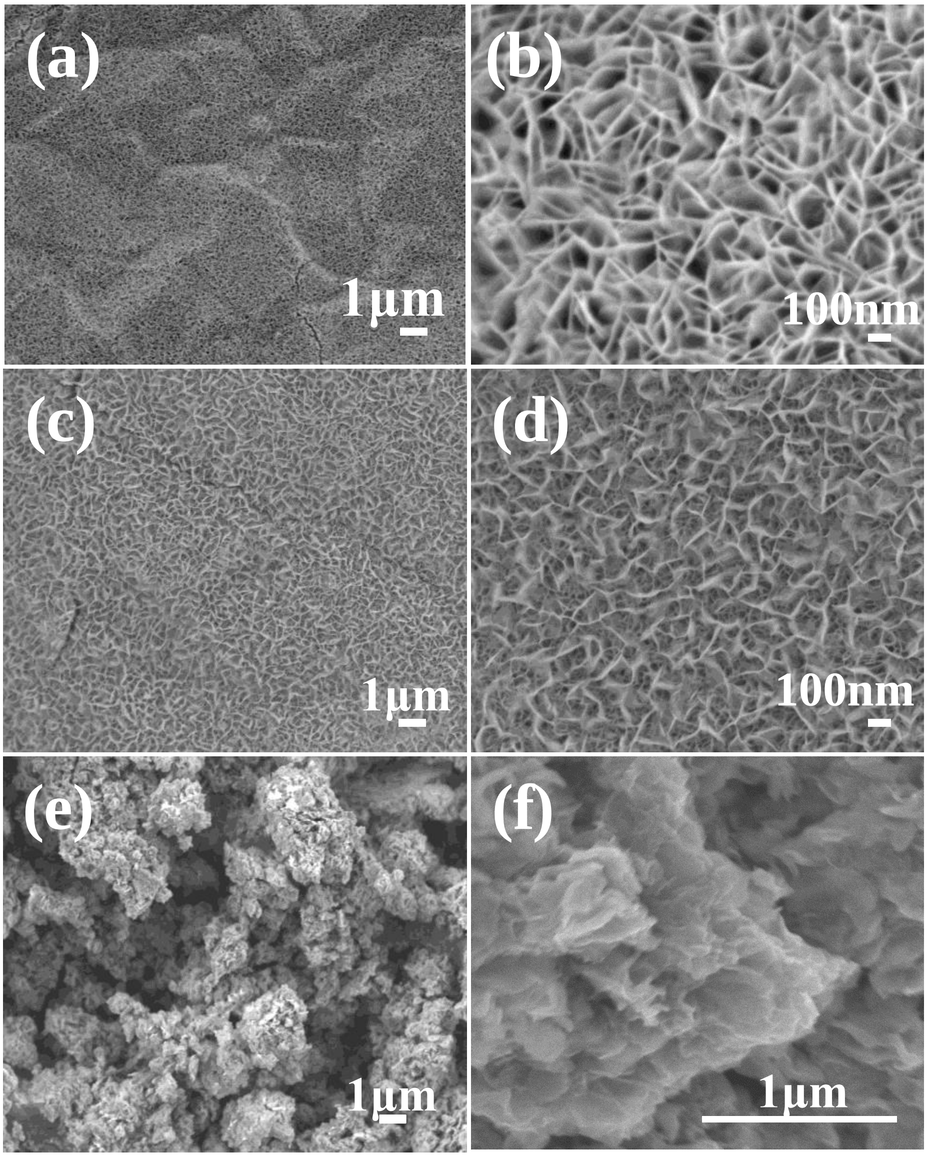


**Figure S1.** The SEM images of Ni-Co-S-1(a, b), Ni-Co-S-3(c, d) and Ni-Co-S powder(e, f).

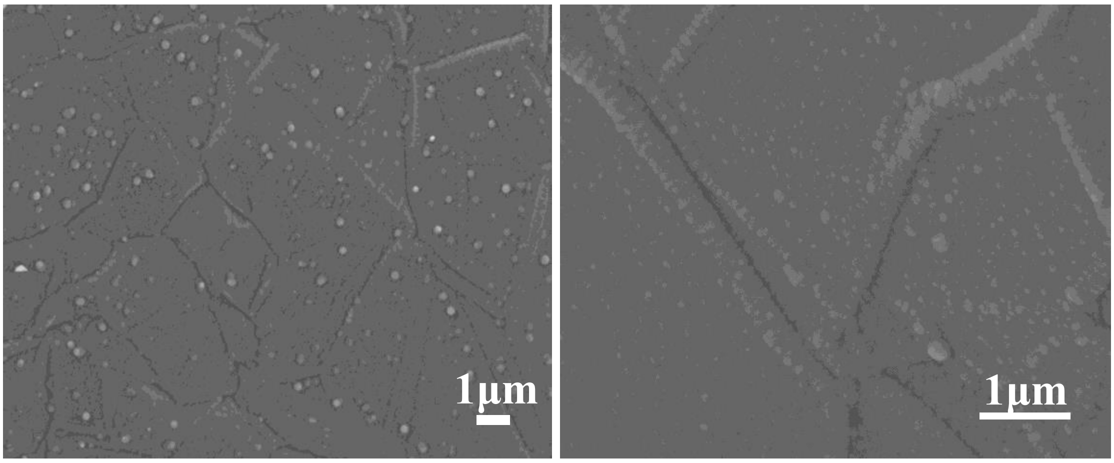


**Figure S2.** The SEM images of PPy/GF.

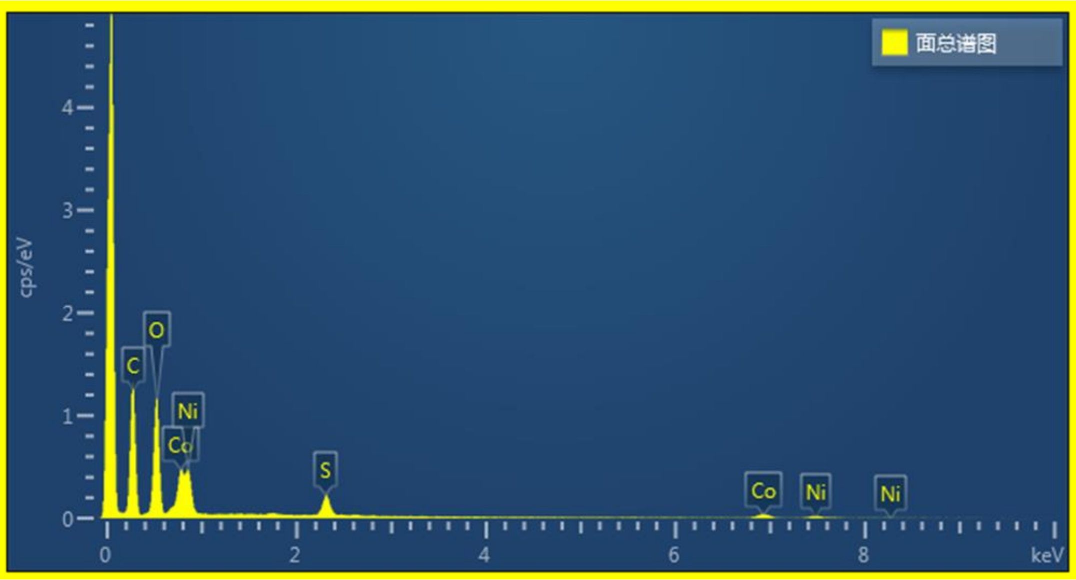


**Figure S3.** EDS spectrum of Ni-Co-S-2/GF

**Table S1** Element content from analyzing EDS spectrum of Ni-Co-S-2/G

| Element | Weight% | Atomic% |
|---|---|---|
| C | **34.60** | **59.6** |
| O | **17.37** | **22.5** |
| S | **3.24** | **2.1** |
| Co | **22.14** | **7.9** |
| Ni | **22.65** | **7.9** |

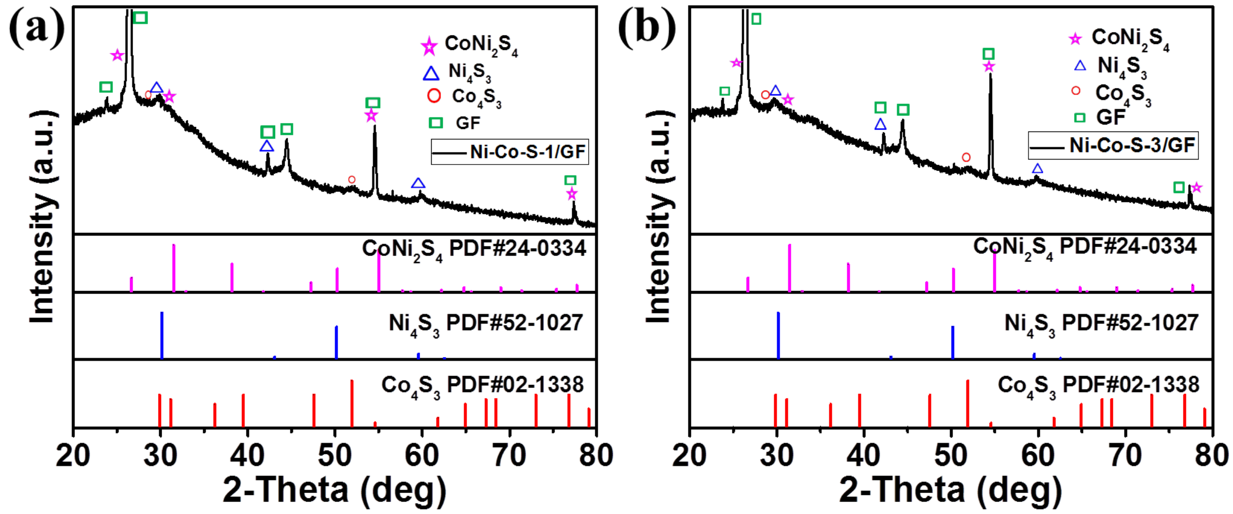


**Figure S4.** The XRD patterns forNi-Co-S-1/GF (a) and Ni-Co-S-3/GF (b).

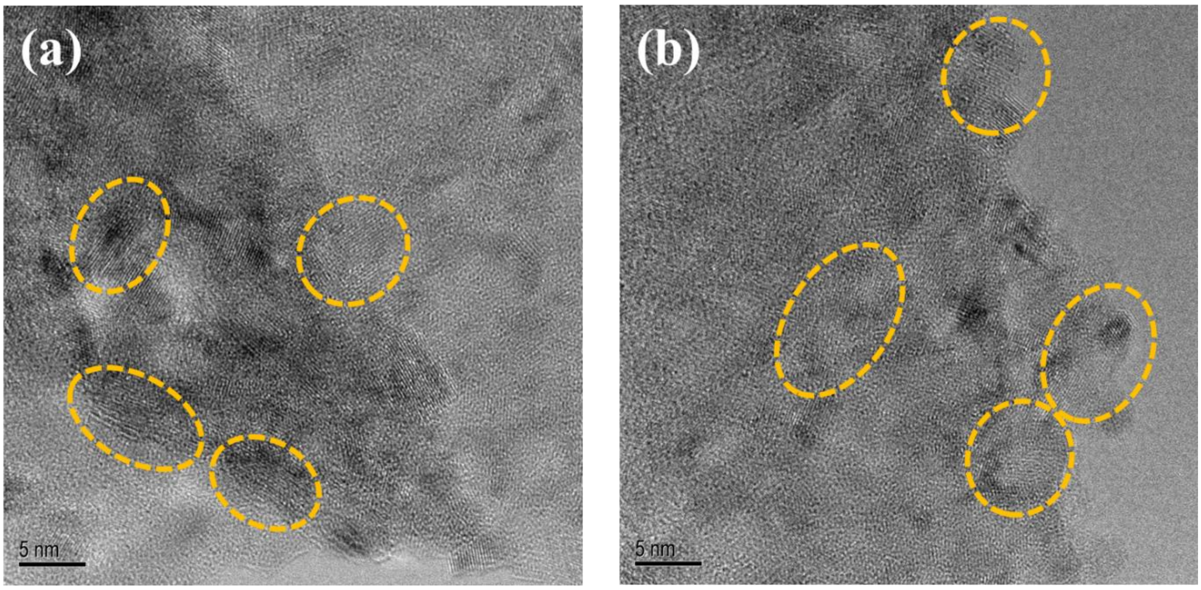


**Figure S5.** TEM images of Ni-Co-S-2/GF

**Synthesis of Ni–Co–S powder**

Ni–Co–S powder was synthesized by a simple one-step hydrothermal method. Moderate RGO was dissolved by ultrasound in 50 mL DI water in 3 neck flask. Then prepared 50ml solution containing 0.1 mmol $Ni(NO_3)_2{\cdot}6H_2O$, 0.1 mmol $Co(NO_3)_2{\cdot}6H_2O$ and 1mmol thiourea. The boiling flask-3-neck was then placed in an oil bath which was pre-heated to

95℃. The prepared solution was added dropwise to the hot solution. The reaction was then carried out under magnetic stirring for 8 h. The product was collected after washing with DI water and further dried.

**Table S2.** Energy densities and power densities for nickel cobalt sulfide-based ASCs reported, recently.

| Positive electrode | Negative electrode | Highest potential | Maximum energy density | Maximum power density | Ref. |
|---|---|---|---|---|---|
| Ni-Co-S/G/NF | PPy/GF | 1.6V | 79.34 Wh $kg^{-1}$ (at 825 W $kg^{-1}$) | 27.73 kW $kg^{-1}$ (at 16.1 Wh $kg^{-1}$) | This work |
| CNTs@Ni-Co-S core/shell arrays | CNTs | 1.6 V | 49.2 Wh $kg^{-1}$ (at 800 W $kg^{-1}$) | 40 kW $kg^{-1}$ (at 18.9 Wh $kg^{-1}$) | [1] |
| $NiCo_2S_4$ mesoporous nanosheets | AC | 1.6 V | 25.5 Wh $kg^{-1}$ (at 334 W $kg^{-1}$) | 8 kW $kg^{-1}$ (at 10.8 Wh $kg^{-1}$) | [2] |
| graphene@$NiCo_2S_4$ nanoparticles | AC | 1.7 V | 68.5 Wh $kg^{-1}$ (at 850 W $kg^{-1}$) | 17 kW $kg^{-1}$ (at 37.7 Wh $kg^{-1}$) | [3] |
| core–shell $NiCo_2S_4$ nanostructures | porous carbon | 1.6 V | 22.8 Wh $kg^{-1}$ (at 160 W $kg^{-1}$) | 2.47 kW $kg^{-1}$ (at 10.6 Wh $kg^{-1}$) | [4] |
| Ni-Co-S ball-in-ball hollow spheres | graphene/ carbon spheres | 1.6 V | 42.3 Wh $kg^{-1}$ (at476 W $kg^{-1}$) | 10.2 kW $kg^{-1}$ (at 22.9 Wh $kg^{-1}$) | [5] |
| porous Ni-Co sulphides | RGO | 1.6 V | 37.6 Wh $kg^{-1}$ (at 775 W $kg^{-1}$) | 23.25 kW $kg^{-1}$ (at 17.7 Wh $kg^{-1}$) | [6] |
| 2D porous Ni-Co Sulfide | AC | 1.8 V | 41.4 Wh $kg^{-1}$ (at 414 W $kg^{-1}$) | 4.8 kW $kg^{-1}$ (at 23.8 Wh $kg^{-1}$) | [7] |
| $NiCo_2S_4$ nanosheets | FeOOH nanorods | 1.6 V | 45.9 Wh $kg^{-1}$ (at 1.7 kW $kg^{-1}$) | 8.6 kW $kg^{-1}$ (at 19.9 Wh $kg^{-1}$) | [8] |
| 3D cauliflower-like $NiCo_2S_4$ architectures | AC | 1.6 V | 44.8 Wh $kg^{-1}$ (at 401 W $kg^{-1}$) | 16 kW $kg^{-1}$ (at 23.1 Wh $kg^{-1}$) | [9] |
| mesoporous $NiCo_2S_4$ nanoparticles | AC | 1.5 V | 28.3 Wh $kg^{-1}$ (at 245 W $kg^{-1}$) | 9.8 kW $kg^{-1}$ (at 6.8 Wh $kg^{-1}$) | [10] |

## Reference


[1] T. Peng, H. Yi, P. Sun, Y. Jing, R. Wang, H. Wang, X. Wang, *J. Mater. Chem. A* **2016**, 4, 8888.

[2] X. P. Zhibin Wua, Xiaobo Jia, Yirong Zhua, Mingjun Jinga, Qiyuan Chena, Feipeng Jiaoa *Electrochim. Acta.* **2015**

[3] Y. Xiao, D. Su, X. Wang, L. Zhou, S. Wu, F. Li, S. Fang, *Electrochim. Acta.* **2015**, 176, 44.

[4] W. Kong, C. Lu, W. Zhang, J. Pu, Z. Wang, *J. Mater. Chem. A* **2015**, 3, 12452.

[5] L. Shen, L. Yu, X. Y. Yu, X. Zhang, X. W. Lou, *Angew. Chem. Int. Ed.* **2014**, 54, 1868.

[6] H. Chen, Prof. D. J. Jiang, L. Zhang, Y. Zhao, D. Guo, Y. Ruan, D. Xia, *Chempluschem* **2015**, 80, 181.

[7] X. Li, Q. Li, Y. Wu, M. Rui, H. Zeng, *ACS Appl Mater Interfaces* **2015**, 7, 19316.

[8] Y. Li, M. Zhou, X. Cui, Y. Yang, P. Xiao, L. Cao, Y. Zhang, *Electrochim. Acta.* **2015**, 161, 137.

[9] Y. Xiao, Y. Lei, B. Zheng, L. Gu, Y. Wang, D. Xiao, *RSC Adv.* **2015**, 5, 21604.

[10] Y. Zhu, Z. Wu, M. Jing, X. Yang, W. Song, X. Ji, *J.Power.Sources.* **2015**, 273, 584.
